# Fetal and Early Post-Natal Mineralization of the Tympanic Bulla in Fin Whales May Reveal a Hitherto Undiscovered Evolutionary Trait

**DOI:** 10.1371/journal.pone.0037110

**Published:** 2012-05-16

**Authors:** Bruno Cozzi, Michela Podestà, Sandro Mazzariol, Alessandro Zotti

**Affiliations:** 1 Department of Comparative Biomedicine and Food Science, University of Padova, Legnaro (PD), Italy; 2 Museum of Natural History of Milan, Milan, Italy; 3 Production and Health, Clinical Section, Radiology Unit, Department of Animal Medicine, University of Padova, Legnaro (PD), Italy; State Natural History Museum, Germany

## Abstract

The evolution of the cetacean skeleton followed a path that differentiated this group from other terrestrial mammals about 50 million years ago [1], and debate is still going on about the relationships between Cetacea and Artiodactyla [2], [3], [4]. Some skeletal traits of the basilosaurids (the more advanced forms of Archaeocetes), such as the expansion of the peribullary air sinuses, dental modification and vertebral size uniformity [5] are maintained and further emphasized also in contemporary odontocetes and mysticetes. Using Dual-Energy X-Ray Absorptiometry here we report that the deposition of bone mineral in fetal and newborn specimens of the fin whale *Balaenoptera physalus* is remarkably higher in the *bulla tympanica* than in the adjacent basal skull or in the rest of the skeleton. Ossification of the tympanic bulla in fetal Artiodactyla (bovine, hippopotamus) is minimal, becomes sensible after birth and then progresses during growth, contrarily to the precocious mineralization that we observed in fin whales. Given the importance of the ear bones for the precise identification of phylogenetic relationship in therian evolution [6], this feature may indicate a specific evolutionary trait of fin whales and possibly other cetacean species or families. Early mineralization of the tympanic bulla allows immediate sound conduction in the aquatic medium and consequently holds potential importance for mother-calf relationship and postnatal survival.

## Introduction

The evolution of the cetacean skeleton followed a path that differentiated this group from other terrestrial mammals about 50 million years ago [1], and debate is still going on about the relationships between Cetacea and Artiodactyla [2], [3], [4]. Some skeletal traits of the basilosaurids (the more advanced forms of Archaeocetes), such as the expansion of the peribullary air sinuses, dental modification and vertebral size uniformity [5] are maintained and further emphasized also in contemporary odontocetes and mysticetes. The morphology and structure of the cetacean skeleton clearly indicates adaptation to life in the water [7], a denser medium in which movements are based on vertical oscillation of the long vertebral column. Elongation of the facial skull, coalescence of the cervical spine, and absence of pelvic limbs, are well known key features of the whale and dolphin structure. Imaging evidenced peculiar features of cetacean bones, including absence of a medullary cavity in the humerus, radius and ulna substituted by an hour-glass shaped trabecular architecture [8], [9]; modification of the mandible to increase sound-receiving properties [10]; presence of atypical gradients of density of the rostrum in deep-divers [11], [12]. These morphological features are considerably different from the equivalent of terrestrial mammals, and constitute a peculiar acquisition of the order.

The ontogenesis of the cetacean skeleton has been studied in dolphin species [13], and only partially in larger baleen whales [14]. We had access to a few fetal and newborn specimens of fin whale *Balaenoptera physalus* (Linnaeus, 1758) in which the ossification of the skull was incomplete and individual bones could be separated. In these specimens, the absolute weight of the tympanic bulla exceeds by far that of any other part of the skeleton.

We therefore decided to approach the study of the tympanic bulla in fetal and newborn fin whales by Dual-Energy X-Ray Absorptiometry (DXA), a commonly used medical method to measure bone mineral density (BMD) in man and animals. As DXA calculates BMD based on surface area, it is not an highly accurate technique to measure the actual bone density, which is mass divided by a volume (expressed as grams/cm^3^); actually, one important confounding effect is bone size, due to the missing thickness value in the formula used to calculate BMD. Nevertheless, DXA technology is by far the most widely used technique for bone density measurements, since it is easy to use, relatively safe and able to provide a significantly high measurement precision and accuracy [15].

The validity of DXA as a method to measure BMD in human fetuses and newborns has been reported since 1992 [16]. Global bone mineral content and density increase steadily during prenatal and postnatal life in relation to developmental stage, body weight and length [17], [18]. An exception was evidenced for the human fetal spine, in which bone mineral content, but not BMD, increase with developmental age [19]. However BMD showed similar trends for each vertebra of the entire column. DXA studies on the BMD of the canine spine and bovine metacarpal bones during growth evidenced a positive correlation among vertebral BMD, age, gender and body weight [20], [21].

**Figure 1:**
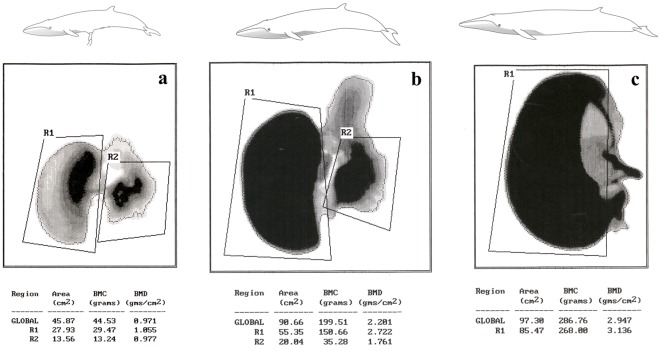
DXA scan reports of the left tympanic bullae of fin whales of different ages. a, 6 months fetus (MSNM Ma7486); b, newborn (MSNM Ma4881); c, adult (MSNM Ma4582), showing the bone mineral content (BMC) and the BMD of the tympanic bulla (R1); periotic bones (R2); whole specimen (GLOBAL).

**Figure 2:**
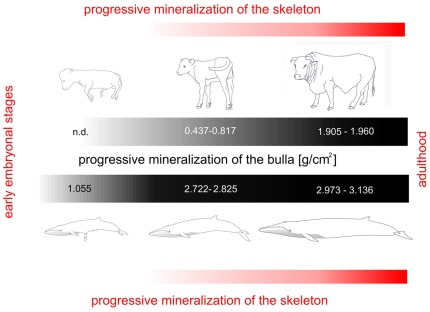
Representation of progressive growth of the tympanic bullae (black bars) and skeleton (orange bars) in bovine and fin whales. The deposition of bone mineral matter starts in the early fetal stages in fin whales, increases steadily in the newborn and achieves comparatively high density in the adult. Bone density in the bovine bulla appears negligible in the early developmental stages and most of the bone mineral content is added in postnatal life.

**Table 1:** BMD values of tympanic bullae (g/cm^2^).

Species	Institution	age	BMD of the bulla/R1
*B. physalus*	MSNM Ma7486	fetus, 6 months (270 cm)	1.055
*B. physalus*	MSNM Ma4881	newborn (545 cm)	2.722
*B. physalus*	MMMTB # 109	newborn (557 cm)	2.825
*B. physalus*	MSNG 46802	adult	2.973
*B. physalus*	MSNM Ma374	adult	3.082
*B. physalus*	MSNM Ma4582	adult	3.136
*B. edeni*	unclassified*	adult	2.603
*B. taurus*	UniPD	newborn, 1 day	0.437
*B. taurus*	UniPD	newborn, 1 day	0.445
*B. taurus*	UniPD	early postnatal, 1 week	0.763
*B. taurus*	UniPD	early postnatal, 1 week	0.774
*B. taurus*	UniPD	early postnatal, 1 week	0.817
*B. taurus*	UniPD	adult	1.905
*B. taurus*	UniPD	adult	1.960
*H. amphibius*	UniPD	early postnatal, <1 month	0.694

All measures refer to the left tympanic bullae. Left-to-right differences in shape, size and density were negligible in our experimental series.

MMMTB = Mediterranean marine mammal tissue bank, University of Padova; Legnaro (PD), Italy; MSNM Ma = Museum of Natural History of Milan; Milan (Italy); MSNGe = Museum of Natural History “G. Doria” of Genova; Genova (Italy); UniPD = Collection of the Faculty of Veterinary Medicine of the University of Padova; Legnaro (PD), Italy.

* = isolated bulla from specimen stranded in extra-national waters, body destroyed; private collector, Milan (Italy).

Specific studies on the tympanic bullae of whales, using Archimede's principle or the Backscattered Electron Imaging method, have already proven that BMD values of the tympanic bulla of adult whales are slightly higher than those of a series of bones from adult terrestrial mammals, including man [22], [23], [24], [25].

## Results

Densitometric scans performed in our laboratory evidenced a specific and differential deposition of bone mineral matter in the bullae of two newborn fin whales (BMDs 2.722–2.825 g/cm^2^), but not in other parts of the temporal complex (BMD 0.729 g/cm^2^), adjacent skull bones (sphenoid bone, BMDs 0.743 g/cm^2^; basal occipital bone, BMDs 0.414 g/cm^2^), or other parts of the skeleton (thoracic vertebra, BMDs 0.743 g/cm^2^; humerus, BMDs 0.692 g/cm^2^) of the same animals. The precocious density of the bone walls of the tympanic bulla in early postnatal fin whales is due to progressive deposition of mineral matter during fetal development, as also indicated by the high mineral content of the same part in a 6 month fetus (BMD 1.055 g/cm^2^; age of the fetus determined using a fetal growth formula based on the body weight [26]). Densitometric scans of the tympanic bullae of fin whales at different ages are represented in Figure 1. Comparative analysis of closely related newborn Artiodactyla revealed minimal density of the bulla (early postnatal *Hippopotamus amphibius*, BMD 0.694 g/cm^2^; newborn *Bos taurus* BMDs 0.437–0.445 g/cm^2^; 1-week old *Bos taurus* BMDs 0.763–0.817 g/cm^2^ – see Table 1) and no differential deposition of mineral matter as compared to adjacent bones or other parts of the skeleton. Density values of the bulla in the adult bovine (BMD 1.905–1.960 g/cm^2^) indicate further mineral deposition during growth. Values of adult *Balaenoptera physalus*, BMDs range between 2.973 and 3.136 g/m^2^; analysis of an adult specimen of the closely related Bryde's whale *Balaenoptera edeni* (Anderson, 1879), yielded similar results, with a BMD of 2.603 g/cm^2^. Details on the bone density values of the tympanic bullae are summarized in Table 1. The progressive deposition of mineral matter in fin whale and bovine tympanic bullae is represented in Figure 2.

## Discussion

Separate tympanic bullae, belonging to unequivocally identified specimens of adult Balaenopteridae, are extremely rare to find, since the bulla in the adult is fused with the rest of the temporal bone and generally displayed in mounted skeletons that are not accessible for densitometric studies. However, a thorough review of the literature and examination of samples available in major Italian Museums of Natural History, indicated that in adult specimens of *Balaenoptera physalus* the density of the bulla increases only by one fourth to one third if compared to newborns. The density of the bulla in adult specimens of terrestrial Artiodactyla progressively increases during postnatal life but – in contrast to what has been observed in *Balaenopteridae* – the rate of mineral deposition is consistent with the rate observed in the adjacent parts of the skull and fully comparable to the rest of the skeleton [21]. Ossification of the occipital complex proceeds faster in the fin whale than in other mysticetes [14], but mineral matter deposition in the bulla apparently occurs earlier than in adjacent parts of the skull or in the rest of the skeleton.

**Figure 3:**
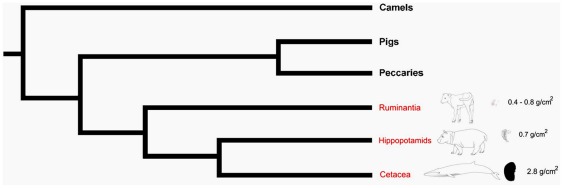
Mineral deposition in the tympanic bullae of newborn Cetacea, Ruminantia and Hippopotamids represented in a simplified cladistic tree. Evolution of Cetacea and Arctiodactyla reflects progressive skeletal adaptation to aquatic/terrestrial life. We hypothesize that fetal and early post-natal mineral matter deposition in the tympanic bulla represents a key adaptive tract for immediate survival in the aquatic environment. Values of BMD, expressed as g/cm^2^ are rounded to the next decimal value.

The absence of a similar early dramatic bone mineral matter accumulation in the tympanic bulla of terrestrial Artiodactyla (Figure 3) suggests an adaptive evolutional trait functional to life in the water in cetaceans. The mechanism of sound transmission to the tympanic bulla in mysticetes is unclear, as they do not rely on the mandibular fat pad as seen in toothed whales to convey the low frequency sounds essential to communication and navigation [10]. A thorough investigation on the density of the fin whale otic bones [23] linked high density to acoustic sensitivity. Since newborn whales can rely only partially on sight, and the importance of smell has been proven only in the bowhead whale but remains debated in other baleen whales [27], sound perception may be their best means for fin whales to keep in touch with the mother and to orient themselves in the aquatic medium, where visibility is limited. Independent of the sound transmission system, immediate functional capacity of the tympanic bulla after birth may help establish mother-calf relationship, perceive and recognize vocalization from members of the family, facilitate learning and survival since the early phases of postnatal life. Thorough investigations on pre- and peri-natal dolphins [28], [29] indicate that perinatal individuals indeed show a clearly ossified tympanic bulla, but no data are available on actual mineral deposition. In our opinion, the dearth of fetal skeletons of Cetacea available for research; the scarcity of separate bones of the basal skull; and the relative rare application of densitometric techniques of investigations to whales and dolphins hindered recognition of this phenomenon in the past.

## Materials and Methods

Each bulla was scanned by means of a DXA device *(Hologic QDR-1000™, Hologic Inc., Waltham, MA, USA)*. During a DXA scan, the X-ray source and detector simultaneously move over the specimens, with the detector measuring the amount of X-rays that pass through the specimen. X-rays of two energy levels are attenuated differently by bone and soft tissue. Consequently, the principle of DXA is based on different X-ray attenuation by different tissues, and BMD is calculated by dividing bone mineral content (measured as the attenuation of X-ray by the bone being scanned) by the area of the site being scanned (expressed as grams/cm^2^).

Each bulla was scanned in a horizontal position, in a caudo-rostral direction and in a dorso-ventral projection. The entire bulla was considered as the region of interest (R1, see Figure 1). All scans were performed by the same operator and the stability of the machine was checked on a regular basis by means of its calibration phantom *(Hologic Calibration Phantom™, Hologic Inc., Waltham, MA, USA)*.

Since defleshed specimens were employed in the present study, a Lexan™ platform was used for examination of the bullae. Lexan support enhances densitometric protocols for small samples [30]. The analysis software of the DXA scan automatically subtracts the mass of the platform from the measured mass of the specimen plus the platform. The resulting difference is the bone mass of the specimen. Thus, by using this platform, filtration of the x-ray beam is optimized and beam-hardening effects are eliminated, resulting in improvement of the linearity of BMD results.
